# The emergency department carbon footprint calculator: Design and validation

**DOI:** 10.1097/MD.0000000000041652

**Published:** 2025-05-30

**Authors:** Lucas Rodriguez-Jimenez, Macarena Romero-Martín, Juan Gómez-Salgado

**Affiliations:** a Sustainability Manager at Central London Community Healthcare NHS Trust, London, UK; b Faculty of Nursing, Universidad de Huelva, Huelva, Spain; c Department of Sociology, Social Work and Public Health, Faculty of Labour Sciences, Universidad de Huelva, Huelva, Spain; d Safety and Health Postgraduate Programme, Universidad Espíritu Santo, Guayaquil, Ecuador.

**Keywords:** carbon footprint, emergency departments, environmental impact, greenhouse gas emissions, life cycle assessment, nursing

## Abstract

Carbon footprint calculators could lead to positive environmental actions, raise awareness, and justify sustainable actions. Emergency departments worldwide receive a large number of patients every year, representing a high carbon footprint. Our tool aims to provide a comprehensive tool created by emergency nurses to measure the carbon footprint of emergency departments. A literature review was conducted to establish the boundaries of the calculator. The pertinence of the resulting boundary inventory was evaluated by a panel of experts. A version 1 of the calculator was designed including the experts’ suggestions. The scope, accuracy of calculation and pertinence of conversion factors were assessed by the same panel of experts. Tool was also tested by a group of health professionals as potential users, in terms of clarity, ease of use, viability, and usefulness. Results: The agreement among experts was measured with the content validity index and Aiken *V*. The results showed good content validity (CVI = 0.74 and *V* = 0.87 in the first panel; CVI = 0.81 and *V* = 0.90 in the second panel). Three main themes emerged from the focus group: calculator overview, data collection and benefits. A validated, and comprehensive tool was obtained for carbon footprint calculation, offering a thorough analysis of scope 1, 2 and 3 emissions in the emergency department.

## 1. Introduction

The impacts of climate change on human health are already evident and they are most likely to cause more harm over the coming years due to its rapid development.^[1]^ July 2023 was registered as the hottest month on record to date, and the average global temperature for the period from January to September 2023 was 0.52°C higher than the corresponding 1991 to 2020 average.^[2]^ Climate change can affect human health due to complex and interrelated mechanisms, including extreme temperature, extreme weather events, increase of air pollution, increase of infectious diseases, lack of food and risk of malnutrition, increase of mental health presentations, and migration, among others.^[1]^ Heat-related deaths in the elderly have increased up to 54% in the last 2 decades, with 61 672 deaths reported only in 2022 across Europe.^[3]^ Extreme weather events, such as wildfires, have caused devastation in Canada, the USA, Algeria, Greece, Spain, Italy and Turkey between 2021 and 2022.^[4]^ Australia registered 450 deaths, 13,000 emergency asthma presentations and a large number of respiratory and cardiovascular admissions, displacing thousands of people and worsening mental health outcomes, after the bushfires occurred in 2020 to 2021.^[5]^ Air pollution has been associated with an increase of diseases such as stroke, chronic obstructive pulmonary disease, trachea, bronchus and lung cancers, exacerbations of asthma and lower respiratory infections, among others.^[6]^ The World Health Organisation (WHO) estimated that 99% of the urban population is exposed to harmful levels of air pollution.^[7]^ Air pollution is responsible for 6.7 million premature deaths annually and data have shown that air pollution decreases life expectancy by 1.8 years worldwide.^[8]^ The incidence rate of infectious diseases such as malaria has increased up to 31.3% in areas of Latin America and 13.8% in the highland areas of Africa, whereas dengue transmission has also risen by about 12% in the same areas from 1951-to-1960 to 2012-to-2021.^[9]^ There has been an increase of food insecurity globally in the last decade, with 720 to 811 million people suffering hunger in 2020.^[10]^ Furthermore, the increased incidence of all these impacts has a significant influence on mental health and human migration.^[11,12]^ Therefore, climate change is not only a climate emergency but also a health emergency.

The term carbon footprint emerges as a necessity to quantify greenhouse gas (GHG) emissions comprehensively. It includes both direct and indirect emissions of GHGs attributable to various processes, products, or organizational activities, and is measured in carbon dioxide equivalent (CO_2_e). These emissions correspond to the 7 GHGs specified by the Kyoto Protocol: carbon dioxide (CO_2_), methane (CH_4_), nitrous oxide (N_2_O), hydrofluorocarbons (HFCs), perfluorocarbons (PFCs), sulphur hexafluoride (SF_6_), and nitrogen trifluoride (NF_3_). Direct emissions refer to those emissions over which an individual or organization holds direct control – for instance, burning fossil fuels for on-site heating or utilizing transportation directly managed by the entity. Indirect emissions refer to emissions related to the activities of the individual or organization but occurring outside their immediate control – examples include emissions from the production of disposable items or the use of purchased electricity, as the emissions are generated elsewhere but attributed to the organization’s activities. The Greenhouse Gas Protocol outlined by the World Resources Institute classifies GHG emissions into 3 scopes: scope 1 emissions or direct emissions such as energy usage, anesthesia gases, or in-house freight transport (excluding purchased electricity) under the organization’s direct purview, scope 2 emissions or indirect emissions linked to purchased electricity or electricity usage sourced from external providers, and scope 3 emissions which include all the other indirect emissions not directly controlled by the organization, including those from supply chains and employee commuting.^[13]^

Methodologies for carbon footprint analysis include bottom-up life cycle assessment – analyzing the materials and processes involved in producing an item, multiplying each by a conversion factor-, top-down economic input–output analysis – utilizing the financial costs associated with a product or process, multiplied by a conversion factor-, hybrid models-combining both bottom-up and top-down approaches, selecting the most suitable depending on data accessibility and availability-.^[14]^

The healthcare sector is responsible for 4% to 5% of greenhouse gas (GHG) emissions.^[15]^ Several studies have assessed the carbon footprint of national healthcare systems,^[15,16]^ whereas others have investigated the carbon footprint in regional^[17,18]^ and local healthcare settings^[19,20]^ The National Health System (NHS) in the United Kingdom (UK), for instance, conducted an extensive evaluation, revealing that its carbon footprint accounted for a substantial 6.3% of the nation’s total emissions.^[15]^ Similarly, Spain and Australia have reported analogous figures, with contributions to national emissions hovering around 5.4% and 7.2%, respectively. Tennison et al delineated the carbon footprint associated with various healthcare activities in the UK.^[15]^ For instance, the utilization of a hospital bed on a daily basis was found to correspond to a significant emission of 125 kg CO_2_e, while outpatient appointments generated 75 kg CO_2_e per visit, and General Practitioner (GP) surgeries contributed 66 CO_2_e per appointment. Studies by Malik et al^[21]^ and Keller et al^[22]^ have identified the substantial contribution of supply chain activities and patient transportation to the overall carbon footprint of healthcare operations. Furthermore, Nicolet et al conducted a carbon footprint analysis focusing on GHG emissions from GP surgeries in Switzerland.^[18]^ Their findings revealed an average emission of 30 tons of CO_2_e annually per GP surgery. A detailed breakdown illustrated that a significant proportion – 45.7% – was attributed to patient and staff transportation, highlighting the considerable impact of mobility-related activities on carbon emissions. Heating constituted another substantial portion, accounting for 29.8% of emissions, followed by consumables (5.5%), courier services (5.8%), and miscellaneous services such as blood analysis and X-rays (1%). In conclusion, these studies underscore the multifaceted nature of GHG emissions within the healthcare sector and highlight the pressing need for comprehensive strategies to mitigate their environmental impact while ensuring the delivery of quality healthcare services.

Nurses and other healthcare professionals play a pivotal role in health promotion, disease prevention, and care delivery. However, healthcare activities significantly contribute to the rapid progression of climate change, which, in turn, will affect public health and undermine some key principles of healthcare professions, such as beneficence and non-maleficence. Hence, healthcare professionals have a responsibility to reduce these emissions while ensuring the safe delivery of care and adherence to professional principles. Furthermore, healthcare professionals can make a significant impact in both reducing climate change effects and supporting communities to adapt to its consequences.^[23]^ Nurses, doctors, and other healthcare professionals are considered some of the most trusted professions worldwide, and hence have a privileged position to advocate for initiatives against climate change.^[24]^ The Intergovernmental Panel on Climate Change (IPCC) has highlighted that sustainable practice can only be possible with the commitment of all professionals.^[25]^

Emergency departments worldwide receive a large number of patients every year; for instance, nearly 136 million patients visit the emergency department annually in the US, while 24.4 million do so in the UK, representing a service of high intensity, which is likely to have a high carbon footprint. The Green Emergency Department (GreenED) project in the UK seeks to transform clinical care and emergency services provision, ensuring that they align with the next zero principles and improve patient’s outcomes.^[26]^ The development of a tool to assess ED carbon footprint could have a significant impact on the development of sustainable initiatives within EDs^[27]^ Carbon footprint calculators could lead to positive actions (e.g., establishing areas of high carbon intensity), raise awareness, justify sustainable actions, and have an influential effect to bring about change.^[28]^ Although there have been several approaches to calculate carbon footprints in ED,^[29,30]^ our tool aims to provide a comprehensive tool created by emergency nurses that then can be used to implement sustainable changes in the emergency department. The aim of this study was to validate the ED carbon footprint calculator, a tool that will help to carry out baseline carbon footprint assessments in EDs, identify areas of high carbon intensity or hotspots, and facilitate the monitoring of sustainable initiatives. This research was conducted between October 2022 and November 2023.

## 2. Materials and methods

**Figure 1.**
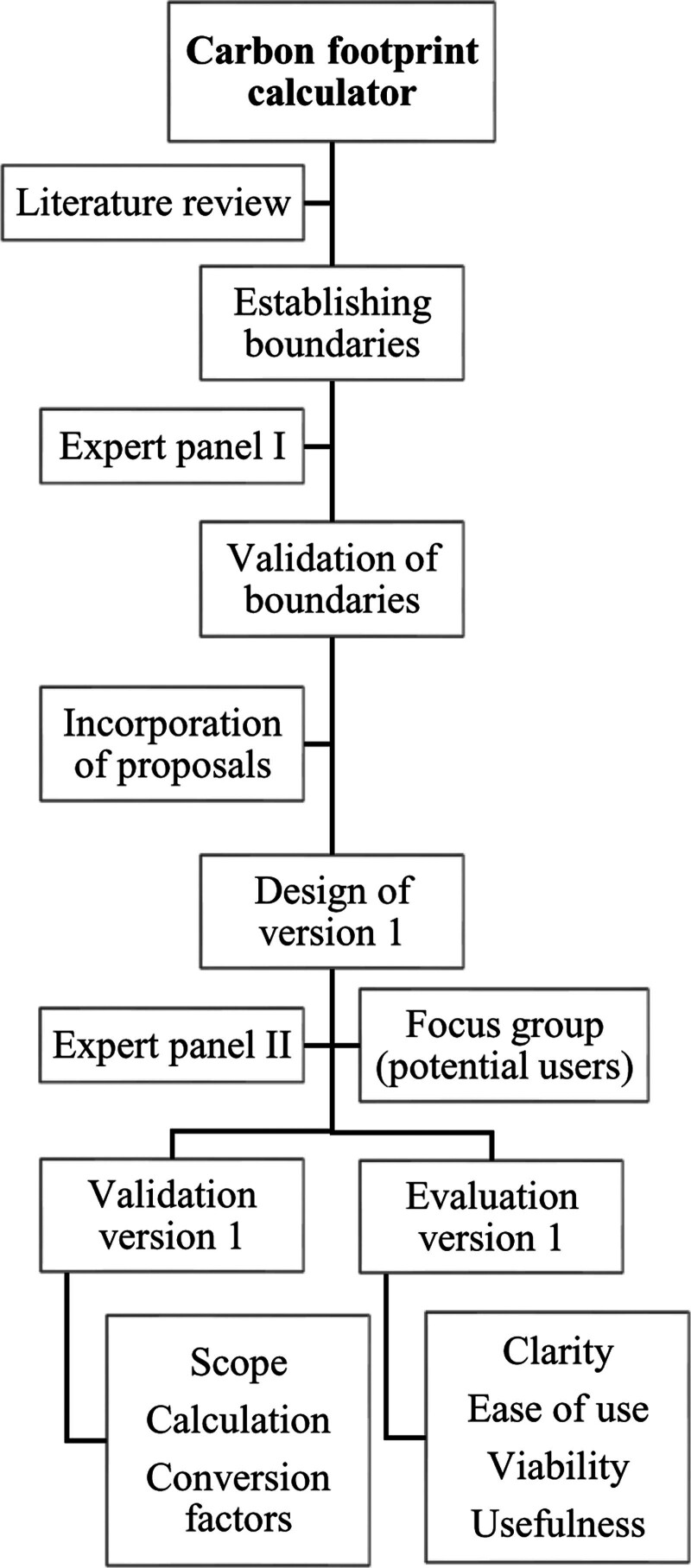
Phases of development and validation of the ED carbon footprint calculator.

The development and validation of the ED carbon footprint calculator followed the phases summarized in Figure 1:

### 2.1. Establishing the boundaries

The first step in designing the emissions calculation tool was to define the boundaries of the measurement. That is, the area to be covered and the information to be included in the data collection and calculations.

#### 2.1.1. Organizational boundaries

The ED of a hospital setting was established as an organizational boundary because it is a core unit with a defined function and a structure of material and human resources that respond to that function. The main purpose of the ED is to deal with urgent and emergency cases that require immediate attention, through rapid diagnosis and the administration of effective medical or surgical treatment in a very short time.^[31]^ The direct and immediate access of patients makes it a highly frequented service by the population.^[32]^ Therefore, emission reduction measures implemented in these units would not only contribute to sustainability but would also have a greater diffusion and social impact.

Another argument for focusing the tool on the hospital emergency department is novelty, as there are previous studies on the carbon footprint of operating theaters,^[33]^ hemodialysis units,^[34]^ intensive care units,^[20]^ ambulance services,^[35]^ geriatric centers,^[36]^ dermatology^[37]^ or radiology,^[38]^ but none have been identified for hospital emergency departments.

#### 2.1.2. Operational boundaries

Once the boundaries of the organization have been defined in terms of the facilities over which it has sufficient management to perform the emissions calculation with the tool (emergency room), the operational boundaries need to be defined. This involves identifying the direct and indirect emissions associated with the processes within the facility and selecting which ones to include in the analysis.

For this purpose, a literature review was conducted to identify the most common categories included in previous carbon footprint assessments in healthcare settings.^[39]^ This literature review led to a boundary inventory that was summarized in a table of contents including 11 categories, as well as information regarding data sources for carbon footprint calculations, key data collected, and units of data for each category.^[27]^ The 11 categories comprised were: energy and heating, anesthetic gases, freight transport, purchased electricity, catering and food, disposables and consumables, medical and nonmedical equipment, pharmaceuticals, transport, waste, and water. Data sources varied depending on the category, although they were mainly invoicing and billing, meters, auditing, and surveys. The key data collected reflected the most important data to be obtained for each category. Our methodology followed the environmental reporting guidelines provided by the Department for Environment, Food & Rural Affairs (DEFRA) from 2019.^[39]^ The conversion factors selected for all the categories in this assessment tool were obtained from the annual report published by DEFRA, except in the category of waste, in which the conversion factors provided by Rizan et al were used.^[33,40]^ Lastly, the unit of measurement for the data collected was chosen in accordance with the data provided by DEFRA and Rizan et al to facilitate its calculation.^[33,40]^

The ED carbon footprint calculator incorporated a hybrid method to quantify carbon emissions in EDs, in which bottom-up and top-down life cycle assessments are used depending on the availability and accessibility of the data.

### 2.2. Validation of the boundaries

The boundary inventory was evaluated by an expert panel to assess its comprehensiveness. The experts were selected by convenience sampling to meet the inclusion criteria: 1) being a healthcare professional, 2) having at least 3 years of experience in EDs, and 3) having at least 3 years of experience in environmental sustainability initiatives or proven experience in carbon footprint calculation. A total of 15 experts were invited, including nurses and doctors, among whom 11 agreed to participate in the study. They were given the boundary inventory, and, through an online questionnaire, they were asked to evaluate the relevance of each item (category, data source, data collection, and unit of measurement) with a dichotomous scale (Yes/No). Moreover, they were invited to make additional valuations and suggestions. Participation in the panel was voluntary.

The content validity, that is, the capacity of an instrument to measure the phenomena which it was designed for, was measured using the content validity index (CVI) for each item and for the entire tool in general. It allowed quantifying the degree of agreement among the experts regarding the relevance of each item.^[41]^ In accordance with the adaptation proposed by Tristán-López for the panels with a reduced number of members, the CVI was considered as acceptable for values above 0.59 in the case of a panel of 11 experts.^[42]^ Additionally, Aiken *V* was also evaluated, which allows assessing the relevance of each item with respect to its construct, taking into account both the number of categories provided to the experts and the number of participating experts. The value may range between 0 and 1, being more relevant the closer it is to 1.^[41]^

### 2.3. Design of the version 1 of the ED carbon footprint calculator

The contributions of the expert panel were incorporated to the version 1 of the ED carbon footprint calculator, which was created using Microsoft Excel for Mac v16.65. This version was divided into different Excel sheets following the next order: introduction, content, emergency department details, scope 1 emissions, scope 2 emissions, scope 3 emissions – divided into 7 sheets to facilitate its navigation – and results. Each Excel sheet contained several sections and items for each category, as well as a cell at the bottom of the sheet with the total amount of carbon emissions for the category. The ED at the Royal Free Hospital in London was selected as a pilot site for the identification and collection of data between November 2022 and February 2023. Data were collected through observation and from hospital records. The tool was designed to make calculations over a period of 1 year.

#### 2.3.1. Introduction, table of contents, and emergency department details

The introduction included comprehensive information regarding the contents and organization of the calculator, as well as the scope and boundaries, the conversion factors used, and the “how to use” and “how to navigate” instructions. A content sheet was also created with hyperlinks to direct the user straight to contents desired and facilitate its navigation. The emergency department details sheet contained different cells to introduce data regarding the department details, such as name of the hospital site, floor space of the emergency department and the hospital premises, number of staff working in the emergency department and number of attendances. The surface of the emergency department and the hospital must be introduced when submeters are not available in the department, as the approach by Prasad et al was applied, according to which the total consumption of the site is multiplied by the department’s surface and divided by the total surface of the site, providing and estimation of the department’s consumption and subsequently its carbon footprint.^[20]^ This approach was utilized in the categories of energy, electricity, and water consumption.

#### 2.3.2. Scopes 1, 2, and 3

An exhaustive compilation of 1095 items was designed, and these were grouped into the 3 scopes proposed by the Greenhouse Gas Protocol.^[13]^ Each scope was organized into categories, as described in Table 1. In the calculations based on the financial expenditure, there was a box to facilitate the general calculation, without detailing the items.

**Table 1:** Design of the version 1 of the ED carbon footprint calculator.

Scope	Category (no. items)	Subcategories	Clarifications
Scope 1	Energy consumption (4)	Burning heating oil	These are the main sources of energy consumption used in hospital premises across the UK according to the ERIC database. The conversion factor included Well-to-Tank, which refers to those emissions related to the extraction, refining and transportation of raw fuel to the hospital site.
		Natural gas	
		LPG	
		Coal	
	Anaesthetic gases (8)	Nitrous oxide (Entonox)	The most commonly anesthetic gases in ED were included. Nitrous oxide was specified into different sizes according to the capacity of each cylinder and the code of each cylinder was provided to facilitate their identification.
		Methoxyflurane (Penthrox)	
	Hospital transport (1)	Ambulance (inter-hospital)	Freight transport was not included, as it is normally controlled by the hospital procurement team rather than by the ED. Inter-hospital transport or transport of patients between different sites or trusts were included, as they represent direct emissions produced by the activity of the ED. It was calculated based on km in a standard diesel ambulance.
Scope 2	Electricity (1)	Electricity	Renewable energies were not included as such, as they are included in the conversion factor provided by DEFRA and the increase of use of those would decrease the conversion factor for electricity.
Scope 3	Disposable/consumables (481)	Airway	Each subcategory has a folder with different items for selection, with a total of 481 articles described. A description is provided, together with the units included in each package and the codes to facilitate the identification of the products. The information is added to the calculator as consumed units. Moreover, blank spaces were integrated to add the products that are not included in the folders. The conversion factor was applied based on the financial cost of medical instruments. The financial cost for each item was obtained from the hospital records at the pilot site and these match the NHS supply chain prices, allowing their applicability to other sites ordering through this platform.
		Breathing	
		Circulation	
		Disability	
		Gloves and PPI	
		Gynae	
		Hygiene and cleaning	
		Housekeeping	
		Immobilisation	
		Specimens	
		Urology	
		Wound care	
		Others	
	Medical equipment (92)	Medical equipment (electrical)	Each subcategory has a folder with different items for selection, with a total of 92 articles described. A description of the article, the manufacturer and the model is provided to identify the units that are added in the calculator, or a similar model, if the article is not available. A list of the medical equipment items was obtained from the hospital records at the pilot site. Information regarding the price, weight and life expectancy of each item was obtained from the information provided by manufacturers. Furniture and medical instruments’ carbon emissions were calculated based on their cost, while electrical and imaging items were calculated based on their weight. The total carbon footprint of each item was divided by its life expectancy, as this calculator was designed to provide the carbon footprint of 1 year.
		Medical equipment (furniture)	
		Medical equipment (medical instruments)	
		Imaging equipment	
	Nonmedical equipment (7)	Nonmedical equipment	A list of the entire nonmedical equipment was obtained from the hospital site, and 7 of the most used items were chosen as representative of this category. Conversion factors for each item were applied based on their weight and the material.
	Pharmaceuticals (407)	Injectables	Each subcategory has a folder with different items for selection, with a total of 407 articles described. The name (in alphabetical order), dose and presentation are provided to facilitate identification. Additionally, blank spaces were integrated to add the products that were not included in the folders. Pharmaceutical items were obtained from the department’s ordering records at the pilot site. Conversion factors based on the financial cost were applied. The price for each item was obtained from the department’s ordering records. Due to the large number of items, we excluded those items that were rarely used and had a cost of less than £1, since their contribution to the overall carbon footprint would be negligible.
		Fluids	
		Oral medication	
		Topicals	
		Nebulisers and inhalers	
		Eye drops	
		TTA pack	
		Enemas and suppositories	
		Diagnostics	
		Nasal medication	
	Transport (50)	Transport	A comprehensive list of 50 transport methods available in DEFRA (2022) was obtained, including different types and sizes of cars – such as petrol, diesel, hybrid and electric, and small, regular and large –, motorcycles, active traveling – such as walking or cycling – and public transportation – bus, tube and train. We also added a key at the bottom of the sheet explaining the engine sizes corresponding to small, medium and large vehicles. The conversion factor was calculated per km in private vehicles, whereas, for public transportation, km per passenger was applied.
	Waste and water (12)	Waste	Eleven different waste types were included. Conversion factors were applied based on the weight of the waste. Laundry services were excluded, since this is a process external to ED; moreover, due to the lack of nonspecific conversion factors available for this activity, the data are inaccessible.
		Water	
	Catering and food (32)	Meals	A list for 32 items regularly ordered by the pilot site was created, which included their weight. We introduced a blank cell in weight for a regular meal or sandwich as weight might differ among sites. The conversion factor based on weight from DEFRA (2022) was chosen, as it would provide more accurate data, since NHS sites do not always share the same ordering system for catering.
		Beverage	
		Breakfast	
		Tableware	
		Pediatric food	
		Others	

ERIC = Estates Return Information Collection.

#### 2.3.3. Carbon footprint outcome

The outcome sheet provided a summary of the carbon emissions for scope 1, 2 and 3, as well as a breakdown of the categories of scope 3. The calculator can also provide 2 diagrams with the total carbon emissions and total scope 3 emissions for the emergency department.

The ED carbon footprint calculator includes all the areas where emergency care is provided at the hospital site—such as Minor, Major, Resus or ambulatory services for adults and/or pediatrics. Furthermore, the imaging department that is used by the emergency team and ED administration offices is also included. However, building infrastructure, support services and other diagnostic services – e.g., laboratories and other imaging departments such as nuclear medicine or ultrasound departments – are not included.

### 2.4. Validation of the version 1 by an expert panel

The content validity of the version 1 of the tool was evaluated by an expert panel. In this case, since the relevance of the items had already been assessed by the same group of experts, the evaluation was focused on the reach of the tool, the calculations, and the conversion factors. The same procedure was followed, inviting the 11 experts from the previous panel to participate in the second panel, among whom 8 experts agreed to participate. In this case, as the number of experts is reduced to 8, the minimum acceptable value of the CVI according to the literature was 0.75.^[42]^

### 2.5. Evaluation of the version 1 by a focus group of potential users

Once the suggestions from the panel of experts II had been taken into account, the face validity (or logical association of the items with the concept to be measured) and the presentation and usability of the tool were evaluated by a focus group. The focus group allowed exploring the experiences of the participants about the use of the tool, providing information from the perspective of the user in terms of its clarity, ease of use, viability and usefulness. We ensured that the participants met the profile of the future user of the calculator. To this end, the following inclusion criteria were established: 1) being a healthcare professional, 2) working in an ED, and 3) having no specific experience or knowledge in carbon footprint. A participation invitation was sent to 8 people who met the profile, along with the version 1 of the ED carbon footprint calculator, 2 weeks before the date set for the focus group, in order to have enough time to manage and test it. Lastly, a virtual focus group was held with 3 participants who accepted the invitation. The focus group was recorded and transcribed verbatim for subsequent analysis. One focus group was held, as according to the study conducted by Guest et al on focus group sample size and thematic saturation, 84% of themes were generated by 1 to 3 focus groups, with 4 people able to generate accurate and reliable information.^[43]^ It was moderated by a member of the research team. Participants were selected by convenience. A call was made to ED staff at the Royal Free Hospital, with 4 reminders.

The discussion was developed around the following questions proposed by the moderator: How was your experience testing out the ED carbon footprint calculator? How did you find having instructions on “how to use the calculator” in the first excel sheet? How does it influence the presentation of the calculator? How did you feel while filling out the data? What use can have the calculator in your professional practice?

The content analysis was performed by 2 researchers independently, according to the recommendations of Graneheim and Lundman and their results were compared to reach consensus, requesting the intervention of a third researcher to resolve any discrepancies, although this was not necessary.^[44]^ Firstly, several comprehensive readings of the discourses were performed, in order to familiarize with the content and identify the units of meaning. Then, following an inductive approach in which the categories emerged from the obtained data, we coded the units of meaning, assigning a code to them in line with their content. The codes were classified, compared and grouped based on their similarities and differences, in order to generate the categories. Thus, the content groups that shared common elements were gathered in the same category, enabling the identification of the themes that emerged from the content analysis.

### 2.6. Ethical considerations

The recommendations of the UK Medical Research Council, the authoritative body guiding our study, were followed. At the outset of our investigation, we requested guidance from the NHS Health Research Authority regarding the necessity of ethical approval. Due to the nature of our study and the participation of experts acting in their capacity as healthcare professionals to provide their opinion on the tool, with the aim of conducting an analysis from their professional standpoint rather than about them or for them, approval from the NHS ethics committee was deemed unnecessary. This has been considered an active partnership between the professionals and researchers to influence and shape our proposed tool. Furthermore, in the focus group, no opinions of a private nature were solicited, nor were lived personal experiences explored to investigate a subjective phenomenon.

Nevertheless, the research was conducted in adherence to ethical quality standards. Regarding the expert panels, participants were selected based on their level of expertise and knowledge in the field. They were provided with prior information on the purpose of the study, methods, the role proposed for them, and what was expected of them. Additionally, they were briefed on the handling and custody of data. The database could not be anonymized as we required knowledge of the origin of improvement comments for potential clarifications. Nonetheless, it was strictly confidential, overseen by one of the researchers, and shared exclusively for peer analysis. Furthermore, participants were given the opportunity to withdraw from the study at any point or request the removal of their comments. Experts were informed of these options and willingly, voluntarily, and disinterestedly agreed to participate (evidence of this process could be provided upon request). Informed consent was requested and obtained from all participants.

Regarding the participants in the focus group, they were similarly informed in advance, under the same terms as the experts. They provided informed consent through a declaration. Regarding the recording of the focus group, verbal permission was sought at the outset of the recording, and all participants consented. The video was securely held and transcribed by one of the researchers, with content analysis conducted confidentially by pairs. In manuscript writing, anonymity of the collected data and opinions has been maintained without providing participant-identifying information (evidence of this process could also be provided upon request).

## 3. Results

### 3.1. Validation of the boundaries by expert panel I

The participants in the expert panel I had an average age of 38.0 (SD = 5.4) years, an equal number of males and females, mainly Caucasian ethnicity (63.6%), PhD (90.9%) and MSc (54.6%). All experts were members of the Green ED of the Royal College of Emergency Medicine. The sociodemographic characteristics of expert panel I are described in Table 2.

**Table 2:** Sociodemographic characteristics of participants.

				M (SD)	n	%
*Panel expert I (prototype assessment*)						
Age				38.0 (5.4)		
Gender		Female			5	45.5
		Male			5	45.5
		Prefer not to disclose			1	9.0
Ethnic identities		Caucasian			7	63.6
		Asian			1	9.1
		Indian			1	9.1
		Chinese			1	9.1
		Prefer not to disclose			1	9.1
Healthcare profession		Doctor			10	90.9
		Nurse			1	9.1
Years of experience ED				10.4 (4.6)		
Highest degree		Bachelor degree			3	27.3
		MsC/MA			6	54.6
		MRCEM			1	9.1
*Panel expert II (first version assessment*)						
Age				38.5 (5.6)		
Gender		Female			4	50.0
		Male			4	50.0
		Prefer not to disclose			0	0
Ethnic identities		Caucasian			6	75.0
		Asian			1	12.5
		Indian			0	0
		Chinese			1	12.5
		Prefer not to disclose			0	0
Healthcare profession		Doctor			8	100.0
		Nurse			0	0
Years of experience ED				10.7 (3.4)		
Highest degree		Bachelor degree			3	37.5
		MsC/MA/MRes			5	62.5
		MRCEM			0	0
*Focus group*						
Code	Gender	Age	Highest degree		Health profession	Years of experience ED
P1	Woman	37	Bachelor degree		Nurse	7
P2	Woman	36	MsC/MA		Nurse	9
P3	Woman	40	Bachelor degree		Doctor	14

ED = Emergency Department, M = mean, MA = Master, MRCEM = Royal College of Emergency Medicine, MRes = Master in Research, MsC = Master in Sciences, SD = standard deviation.

In general, the relevance of the content of the prototype was good, with a total CVI of 0.74 and Aiken *V* of 0.87. Specifically, 6 items obtained a CVI below the acceptable value, as shown in Table 3. The lowest values of Aiken *V* corresponded to the measurement units for catering/food (0.55) and disposable/consumables (0.64). The results of the consensus on the relevance of the items of the prototype are summarized in Table 3.

**Table 3:** Results from experts panel I.

		Appropriate n (%)	CVI	*V* Aiken
Energy/heating	Category	9 (81.8)	0.64	0.82
	Data source	9 (81.8)	0.64	0.82
	Data collection	9 (81.8)	0.64	0.82
	Unit	10 (90.9)	0.82	0.91
Anaesthetic gases, metered dose inhalers and other medical/surgical gases	Category	9 (81.8)	0.64	0.82
	Data source	9 (81.8)	0.64	0.82
	Data collection	8 (72.8)	0.45	0.73
	Unit	10 (90.9)	0.82	0.91
Electricity	Category	9 (81.8)	0.64	0.82
	Data source	11 (100.0)	1.00	1.00
	Data collection	10 (90.9)	0.82	0.91
	Unit	11 (100.0)	1.00	1.00
Catering/food	Category	9 (81.8)	0.64	0.82
	Data source	9 (81.8)	0.64	0.82
	Data collection	9 (81.8)	0.64	0.82
	Unit	6 (54.5)	0.09	0.55
Disposable/consumables	Category	8 (72.8)	0.45	0.73
	Data source	11 (100.0)	1.00	1.00
	Data collection	10 (90.9)	0.82	0.91
	Unit	7 (63.6)	0.27	0.64
Medical equipment	Category	9 (81.8)	0.64	0.82
	Data source	10 (90.9)	0.82	0.91
	Data collection	10 (90.9)	0.82	0.91
	Unit	8 (72.8)	0.45	0.73
Nonmedical equipment	Category	9 (81.8)	0.64	0.82
	Data source	10 (90.9)	0.82	0.91
	Data collection	11 (100.0)	1.00	1.00
	Unit	8 (72.8)	0.45	0.73
Pharmaceuticals	Category	9 (81.8)	0.64	0.82
	Data source	10 (90.9)	0.82	0.91
	Data collection	10 (90.9)	0.82	0.91
	Unit	9 (81.8)	0.64	0.82
Transport	Category	9 (81.8)	0.64	0.82
	Data source	10 (90.9)	0.82	0.91
	Data collection	10 (90.9)	0.82	0.91
	Unit	11 (100.0)	1.00	1.00
Waste	Category	9 (81.8)	0.64	0.82
	Data source	9 (81.8)	0.64	0.82
	Data collection	11 (100.0)	1.00	1.00
	Unit	10 (90.9)	0.82	0.91
Water	Category	9 (81.8)	0.64	0.82
	Data source	11 (100.0)	1.00	1.00
	Data collection	9 (81.8)	0.64	0.82
	Unit	10 (90.9)	0.82	0.91
	Total		0.74	0.87

CVI = content validity index.

The comments for improvement provided by the experts and the resulting changes are shown in Table S1, Supplemental Digital Content, https://links.lww.com/MD/P5. In general, 6 experts highlighted the difficulty of specifically calculating the cost of energy, electricity and water for the ED service, since the billing or meters usually perform a calculation of the total consumption of the hospital. In response, the Prasad et al approach was applied, according to which the total consumption of the site is multiplied by the department’s surface and divided by the total surface of the site. This formula can provide an estimation of the department’s consumption and subsequently its carbon footprint.^[20]^ Some comments questioned the aptitude of the measurement units for energy, anesthetic gases, catering/food, medical equipment and nonmedical equipment, as they are neither the most common nor easily obtained. Therefore, the units of the data collected were checked and modified in accordance with the conversion factors provided by DEFRA in order to facilitate the calculations.^[40]^ As suggested by the experts, the brand names of the anesthetic gases have been replaced by their generic names, for example, from Entonox to nitrous oxide. One of the experts suggested including anesthetic gases for pipelines, but the research team decided not to consider it, as anesthetic gases are not commonly used in EDs and, when used, are usually managed by the anesthesia team rather than the emergency physician. The experts expressed their concern about the data-gathering method in the categories of transport, waste and disposable/consumables, due, in the latter case, to the wide variety of materials comprised in it. We acknowledged that data collection for these categories in particular could be challenging. For the disposables/consumables category, 5 different sections were created to facilitate the classification of the data. For the other categories, both cross-sectional studies, an audit for waste and a survey for transport were proposed. Moreover, the experts suggested to include the consumption of renewable energies. It was decided not to include the use of green energies in the calculator, as they are already applied in the conversion factors provided by DEFRA.

### 3.2. Validation of the version 1 by experts panel II

The second expert panel had an average age of 38.5 (SD = 5.6) years, mainly Caucasian doctors (75.0%) and MSc (62.5%). The sociodemographic characteristics of expert panel II are described in Table 2. In general terms, the experts valued the version 1 as relevant, with a total CVI of 0.81 and Aiken *V* of 0.90. Discrepancies were detected regarding the relevance of the calculations and conversion factors of scope 3, with CVI = 0.50 and *V* = 0.75, as shown in Table 4.

**Table 4:** Results from experts panel II.

		Appropriateness n (%)	CVI	*V* Aiken
Scope 1	Comprehensive and representative	8 (100.0)	1	1
	Calculations	7 (87.5)	0.75	0.88
	Conversion factors	7 (87.5)	0.75	0.88
Scope 2	Comprehensive and representative	8 (100.0)	1	1
	Calculations	8 (100.0)	1	1
	Conversion factors	7 (87.5)	0.75	0.88
Scope 3	Comprehensive and representative	8 (100.0)	1	1
	Calculations	6 (75.0)	0.50	0.75
	Conversion factors	6 (75.0)	0.50	0.75
	Total		0.81	0.90

CVI = content validity index.

The comments for improvement suggested by expert panel II and the resulting changes are shown in Table S2, Supplemental Digital Content, https://links.lww.com/MD/P6. With regard to the measurement units, 2 experts suggested revising the units applied in the cells of total fields and conversion factors, so they were checked and corrected. One of the experts pointed out that some items may have been excluded from the category of consumables and disposables. Further to this comment, a black cell in the disposables/consumables category was added for the user to be able to introduce items not available in the calculator. Furthermore, the experts recommended adding a cell to calculate the footprint of the category of disposables and consumables based on the total expenditure instead of doing so item by item, in order to facilitate the calculation for departments in which a thorough calculation cannot be performed. This suggestion was incorporated by including an overall spend field for pharmaceuticals and disposables, given the user option to measure carbon footprint based on the overall expenditure. One of the experts asked whether the costs obtained from the Royal Free Hospital were in line with those of the NHS supply chain catalogue in the category of disposables and consumables, which would question their applicability in other hospitals. Thus, it was compared and confirmed that the prices obtained from the Royal Free Hospital were the same as the prices provided by the NHS supply chain catalogue. Three experts recommended replacing “quantity measured” with “quantity used,” since the quantity measured in the department may not coincide with the quantity used, so it was changed. The experts pointed out calculation errors in the category of anesthetic gases and waste and recommended adding transport and distribution (T + D) and Well-to-tank (WTT) emissions to the category of energy, as well as WTT emissions to the category of transport. These considerations were taken into account in the conversion factors.

### 3.3. Evaluation of the version 1 by the focus group of potential users

The sociodemographic data of the participants are described in Table 2. The analysis of the discourses generated a total of 72 units of meaning, which were grouped in 14 categories (Table 5). Three themes emerged from the content analysis: calculator revision, data collection, and benefits.

**Table 5:** Results from focus group content analyze.

Theme	Category	Quote	
Calculator review (n = 25)	Positive evaluation (n = 2)	*It’s a very thorough and very good tool to be honest.*	P1
	Easy to use (n = 6)	*I think it’s very easy to use. It’s very obvious on how to use it.*	P1
		*Considering I’m not particularly IT literate, I thought it was OK. I didn’t have any major issues with it.*	P3
	Friendly (n = 4)	*I think is friendly enough. I like the fact that you can do the plus and minus. So it’s not too much if you’re not wanting to go into a category.*	P1
		*It’s easy to read language. I like when things are kind of bullet pointed or put out nice and clearly… It looks like it was all nice, kind of clearly little boxes*	P3
	Clear information (n = 4)	*I don’t think it was missing anything (instructions)….I thought it had all the information that it needed.*	P3
		*Everything’s very clearly kind of documented at the start... It’s sort of clearly states what it’s for.*	P3
	Comprehensive data (n = 4)	*It’s a lot to put in, but it’s all relevant. It’s all things that you need to look like.*	P1
		*I don’t think there is anything I thought it was missing (data).*	P2
	Technical (n = 5)	*When I put the quantity per pack, it needs to put a pound symbol*	P1
		*I like that you have the cake symbols in the last tab with the amounts of each category.*	P1
Data collection (n = 16)	Time consuming (n = 3)	*You just need to have the time and know you’re getting into a very thorough process and that’s it.*	P1
	Data easy to obtain (n = 5)	*Asking states for water and electricity consumption should be easy for instance.*	P1
		*There’s probably a nice easy record of like what’s brought in and how often we need to repurchase it and that sort of thing. And you can easily work things out.*	P3
	Data difficult to obtain (n = 8)	*I think the more difficult one to be honest, probably with the transport part because you have to involve patients and ask very specific questions.*	P1
		*Waste is just a nightmare because there’s no record of how much waste we generate… The bags are not used properly anyway. So, what we would document as recyclable waste, a lot of the times is getting incinerated because it’s been contaminated with clinical waste.*	P3
Benefits (n = 31)	Monitoring and comparison (n = 6)	*I think once you have something to compare, it’s a lot…at least you can see if you’re making a difference.*	P1
		*Knowing where we could make changes and where we’re sort of generating our biggest carbon footprint*	P3
		*It’s gonna be useful going forward to kind of monitor the progress that we’re making.*	P3
	Rise awareness (n = 7)	*If we were able to show that everybody making small decisions actually has an effect, that’s cat can only encourage people more to make those little small changes and then hopefully you’d have a little bit of a snowball effect.*	P3
		*Climate change is a big factor in patients health and at will going forward probably be a big factor in some of the patients that we’re seeing. So I think it’s important that we get involved*	P3
	Motivation (n = 6)	*if you’re able to show these are the changes we’ve made and this is the difference it’s made, then that’s going to motivate people a little bit more.*	P3
		*People love to compete and say we are better than others, so that could be a good way to use it (for motivation).*	P1
		*I think a lot of healthcare professionals and people who work in the health industry are also quite passionate about things that concern climate change.*	P3
	Promote change (n = 11)	*If you don’t have quantifiable data, then it’s really hard to kind of make your argument and to push for changes… It might change their practises more if you’re able to kind of hit them with some hard data*	P3
		*…can also help make changes on those things are outside of our control. Because if people compare the carbon footprint on the similar size… and there’s a big difference between the two of them. You can look at why that is so different and what can you do better on the one that’s wasting way too much. And that can be something to use to push those changes from evolve.*	P2
	Decision making (n = 1)	*that promotes or encourages those in charge who are making decisions beyond our control to actually get involved with it and trying to make changes.*	P3

n = frequency of semantic units, P1 = participant 1, P2 = participant 2, P3 = participant 3.

#### 3.3.1. Calculator overview

This theme refers to the experiences with the use of the calculator. The participants valued the tool in a very positive manner, describing it as easy to use, friendly, clear, useful and with a good layout. It included comprehensive data and clear information on how to use it, with detailed instructions. They also reported some technical issues, such as displaying errors or wrong measurement units in some of the cells; that were addressed and fixed.

#### 3.3.2. Data collection

This theme summarizes the opinions on the information required to estimate the footprint and the concerns expressed by the participants about the data collection. In general, the experts stated that the data collection was complex and time consuming, due to the large amount of data required. However, they also recognized that the data were relevant, comprehensive and feasible. They identified the most easy-to-gather data, that is, those with an available record, such as billing or stockage (e.g., disposables/consumables, pharmaceuticals). On the other hand, the data without records, those that are managed outside of the ED department (e.g., waste), and those that involve demanding third parties with a survey (e.g., transport) were perceived as difficult to obtain and a possible cause of bias.

#### 3.3.3. Benefits

This theme gathers the comments of the participants about the usefulness of the calculator and the implications for the clinical practice.

##### 3.3.3.1. Monitoring and comparison

The participants stated that the calculator provides quantitative data that allow making an initial diagnosis and identifying the starting point of the unit and the areas of greater emissions. These quantitative data also allow monitoring the processes, by comparing the data and quantifying the reduction of the footprint of each intervention.

##### 3.3.3.2. Raising awareness

Another usefulness identified by the focus group was awareness, since measuring the emissions helps professionals to visualize the environmental impact of their professional activity. The participants also pointed out the need to raise the awareness of the entire team, since this is a common problem in which all participants must be involved. The awareness generated by this calculator can be contagious and trigger a snowball effect that results in a shared commitment. The participants recognized that climate change is an important factor for human health, and thus, as professionals, they must engage in its approach.

##### 3.3.3.3. Motivation

In the conversation, another benefit emerged as the motivation of professionals toward more sustainable practices. Although healthcare professionals are a sensitive workforce committed to climate change, quantifying the improvements would motivate them to persist with initiatives for the reduction of emissions. The participants also suggested that the calculation of the carbon footprint could be used as an item to evaluate the department and compare it to others, as in a competition.

##### 3.3.3.4. Promoting change

The analyzed discourses underlined the capacity of the calculator to promote changes. It was considered as a valuable tool to lead environmental initiatives. Quantifying data will provide evidence and arguments to persuade health professionals to change their practice, encourage them to maintain the new approach and convince others to join them. This tool can challenge professionals to make changes and realize they are accountable for their actions, as daily practice choices can have a significant impact on carbon footprint reduction.

##### 3.3.3.5. Decision making

The participants recognized the need to involve the management teams, and they pointed out the usefulness of the tool to motivate the making of more sustainable decisions.

## 4. Discussion and conclusions

This study aimed to develop and validate the ED carbon footprint calculator. As a final result, a valid, exhaustive, clear and easy-to-use tool was obtained, which will allow estimating the CO_2_ emissions generated by the activity of hospital emergency services. This carbon footprint calculator has been designed to be used in the UK, however adaptation and use in other countries could be possible by adjusting the items included.

Regarding the validation of the boundaries by expert panel I, the lowest CVI and Aiken *V* values were scored by measuring units used in the categories catering and food categories, disposable and consumables, medical and nonmedical equipment. The units of measurement in the category of catering and food scored the lowest CVI and Aiken *V* values, indicating that the experts did not agree with the units selected or did not find them relevant. So, we replaced number of meals with weight as recommended, which is also in line with the units provided by DEFRA to calculate the carbon footprint of food and drinks.^[39]^ Disposables and consumables had low CVI and Aiken *V* scores for the unit of measurement, which is due to the complexity and extension of this category. The participants also questioned the feasibility of obtaining data regarding waste, although previous studies have shown that it is possible to weigh hospital waste over a period of time and extrapolate these data to the rest of the year.^[45]^ For medical equipment and nonmedical equipment, the panel of experts recommended changing the unit of measurement from kilogram to financial cost—which were reflected with low CVI (0.45 and 0.45, respectively); however, DEFRA provides conversion factors based on weight for electronic equipment, and for nonmedical equipment based on the material they are made of.^[40]^ Furthermore, manufacturers provide the weight and life expectancy of their products, thus we applied the carbon footprint calculation based on items’ weight and life expectancy, as previously applied by Nicolet et al.^[18]^ Nevertheless, medical instruments’ carbon footprint was calculated based on their financial cost, as the weight for each item was inaccessible to the research team.

The results of the validation of the version 1 by Expert Panel II showed disagreement regarding the category of consumables and disposables, as some items may not have been listed in our calculator, which explains the lower CVI and Aiken *V* scores obtained. Therefore, we included blank cells for the user to introduce the item, price and quantity with automatic application of the conversion factor. Furthermore, we added the possibility for the user to introduce the total consumption of energy, electricity and water when sub-meters were available, as well as the option to calculate carbon footprint for pharmaceuticals and disposables and consumables based on the total expenditure. We ensured that the prices obtained at the Royal Free Hospital for disposables and consumables were the same as those available at the NHS supply chain catalogue to enable a wider use of the tool.^[46]^

The focus group evaluated the ED carbon footprint, and 3 main themes were highlighted: calculator’s revision, data collection and benefits. The participants emphasized that the tool was easy to use, thorough and useful. These opinions coincided with the international survey that Collins et al carried out with 4 245 respondents, which found that a personal carbon footprint calculator was considered a valuable tool for 91% of their participants.^[47]^ Regarding data collection, when they were asked about the appropriateness of the items and how feasible it would be to obtain the data, the participants found that data that are on record should be easier to obtain, whereas others, such as those of transport and waste, which require survey or direct auditing in the field, would be more challenging. Transport was emphasized as one of the most challenging categories, requiring a large sample of participants to be representative; however, previous studies have shown feasibility obtaining these data via survey, in which data were collected for a period of time and extrapolated to the rest of the year.^[19]^ Waste was also considered to be very challenging. Previous studies have applied methods similar to the one provided in our calculator for waste data collection, although we recognized the challenges derived from them.^[45]^ Lastly, a number of benefits were expressed, such as promoting change, raising awareness, monitoring and comparison, education, and decision making. The participants stated that measuring the carbon footprint of the emergency department would not only help to raise awareness and promote change among the staff and patients, but it would also encourage stakeholders in decision making. Previous studies have shown that the general population do not act due to their lack of knowledge regarding how their daily activities aggravate climate change, hence the importance of raising awareness to promote change.^[48]^ This would also influence the motivation of the staff and patients to promote change, which is in line with what Collins et al found in a survey were 78% of the respondents felt motivated and inspired with the creation of a carbon footprint calculator.^[47]^ Furthermore, the ED carbon footprint calculator was described as a key tool to monitor and compare carbon emissions, as previously found in other studies.^[49]^ Finally, the participants highlighted the educational role of healthcare professionals amongst other staff and patients and the importance of educating the community to mitigate the effects of human activity on the environment. The use of ecological footprint education has received increasing attention over the last years, and this could have a significant impact on the promotion of change within the community.^[47]^

Regarding the limitations of the present study, it is important to underline those related to the scope of the tool. It is not possible to make an exact calculation of the carbon footprint, since there are either inaccessible data of activity or no appropriate conversion factors. Thus, it is necessary to establish the boundaries of the evaluation in order for the estimation to be as accurate as possible. The reach of this calculator does not include certain areas, such as freight transport and laundry, due to the insurmountable difficulties in their evaluation. Nevertheless, we obtained a calculator that was as exhaustive as possible, with a variety of 1095 items. This list makes the tool more comprehensive, but it also makes it more time consuming. For ease of completion, the items have been grouped into functional categories and subcategories and arranged in alphabetical order.

Another limitation related to the calculation of the carbon footprint is the date of the conversion factor. Although the 2022 report was used in most cases, for some specific calculations it was necessary to employ the 2019 or 2012 reports, due to a lack of availability. Consequently, the calculation is up to date. Moreover, for the calculation of the detailed carbon footprint of pharmaceuticals, the purchase prices of the pilot site Royal Free Hospital were used as reference. The purchase prices may vary among the different trusts according to their commercial contracts. It is estimated that these differences are lower, and, in any case, they would only affect the calculation of the detailed carbon footprint, with no effect on the general calculation of the pharmaceutical expenditure.

With regard to the methodology, another limitation was the small number of participants in the focus group. This reduced the interaction in the group and the capacity to generate discourses. There was also a lack of diversity in the participants in terms of health professionals, as the panel experts were mainly doctors, which may have introduced a bias in the applicability of the tool. However, the focus group participants were predominantly nurses, which may have validated its use by professionals other than doctors.

Despite these limitations, the calculator is presented as a valid tool for the estimation of the carbon footprint in EDs both to evaluate an initial situation and to compare 2 or more scenarios.

This study concludes that the ED carbon footprint calculator is a valid and comprehensive tool, offering a thorough analysis of scope 1, 2 and 3 emissions in EDs, and the possibility of carrying out a baseline assessment, identifying areas of high carbon intensity and monitoring sustainable initiatives. This tool is in line with the zero carbon-emission commitment of the NHS aiming to reduce emissions to 0 by 2045, and it will provide strong evidence to numerous projects carried out in emergency departments, such as the Green ED.

The methodology that we followed to develop and validate the calculator – expert panels 1 and 2 and focus group – generated a comprehensive and robust tool. It was described by users as a valuable, easy-to-use and user-friendly tool. The data presented in the calculator were valued as representative and appropriate, and their collection seems to be feasible for users.

The ED carbon footprint calculator can lead to positive changes in EDs by raising awareness, promoting change, and facilitating the monitoring and comparison of carbon emissions. Furthermore, it can influence staff and stakeholders to carry out major changes in the way healthcare is delivered, as well as to introduce ecological education to other colleagues and staff. This calculator will enable nurses and other healthcare professionals working in the ED to carry out environmental initiatives.

## Acknowledgments

The authors would like to thank the Green ED team from the Royal College of Emergency Medicine and the emergency team at the Royal Free Hospital who collaborated in this study. The authors would also like to specifically express their gratitude to Fran Haynes for all her effort in the data collection and for enabling the development of this comprehensive tool.

## Author contributions

**Conceptualization:** Lucas Rodriguez-Jimenez.

**Data curation:** Lucas Rodriguez-Jimenez, Macarena Romero-Martín.

**Formal analysis:** Juan Gómez-Salgado.

**Methodology:** Lucas Rodriguez-Jimenez, Macarena Romero-Martín.

**Project administration:** Lucas Rodriguez-Jimenez.

**Validation:** Juan Gómez-Salgado.

**Writing – original draft:** Lucas Rodriguez-Jimenez, Macarena Romero-Martín.

**Writing – review & editing:** Juan Gómez-Salgado.

## Supplementary Material




