# Supplementary material for: The emergency department carbon footprint calculator: Design and validation
Source: Medicine (Baltimore). 2025 May 30;104(22):e41652. doi: 10.1097/MD.0000000000041652 (PMC12129524; doi:10.1097/MD.0000000000041652)
Supplement: Supplementary file 1 [file medi-104-e41652-s001.pdf]

Supplementary Table 1. Comments on expert panel I

Category	Comment	Response
Energy/ Heating	With EM in mind will it be possible to really select out the energy used by an individual department with a meter - this is not currently available. Likewise with billing. This is aspirational but not realistic at present? Key data collected but not measurable on an individual level at present where I work so this would be meaningless and not fillable in.	The approach used by Prasad et al. can be applied, according to which the total consumption of the site is multiplied by the department's surface and divided by the total surface of the site. This formula can provide an estimation of the department's consumption and subsequently its carbon footprint.
	You would require all the above information to know the direct expenditure of source 1 emissions for the department, however, I think this may be difficult to achieve as EDs tend to be part of larger building so unsure if you would be able to get separate energy usage for one department alone.	
	I think it is very hard to collect the energy use of the department - it may have to be modelled based on total hospital energy use	
	Might be difficult to isolate Energy Use by Department (unless metering for ED is separate from other departments?)	
	Perhaps explaining the relationship between total hospital Scope 1 emissions calculation and how to figure out what proportion of that is 'used' by the ED would be helpful? Not sure how this calculation would happen, or if it's even possible aside from a very rough estimation.	
	I think these are all things that need to be considered however it may be quite difficult to get the data for the specific use of them for the emergency department as they are normally located in a large building and there may not be a separate meter for their use in the ED. Could we work out a way to calculate the answer based on the size of the department, this would be less accurate but more manageable.	
	For energy - KWh the main way of measuring this in my experience. What does the L refer to? natural gas? - Standard way of measuring is key here - see below. Not sure what the *means in the table? We have a roof full of solar panels - how can this be integrated into this tool - here or next page?3	The conversion factors provided by DEFRA are expressed in these units; therefore, to facilitate the calculations, these units were selected. We decided not to include the use of green energies in the calculator, as they are already applied in the conversion factors provided by DEFRA.
Anaesthetic gases, metered dose inhalers and other medical/surgical gases	"I'm not sure £ is the right measure here. Separating out the various items in the list will give different units. As below MDI - could be in units dispensed as for penthrox. Cost varies between trusts so not directly comparable and getting the cost is difficult. Also, should cost really be a measure when we are talking about environmental topics because things that are green are often not cheaper, but this shouldn't and can't be a block on measurement and implementation. Entonox Litres, Penthrox Units (or cost), Anaesthetic Gases surely Litres? MDI/DPI - units dispensed or used or cost? Going form £ to CO2e is difficult and wouldn't be possible I don't think?"	The units of data collected in this section have been revised and changed. The units of data collected are expressed in litres.
	May want to consider measuring exact numbers of litres used of Entonox. This would also then capture the Entonox used in departments which have manifolds	
	Consistent units of baseline date is essential. in the example above £ in anaesthetic gases doesn't make sense to me -? L of N20 is what we have measured. For Penthrox we have measured units of penthrox used (I.e 1 box = 1 unit) - either having consistent units of data measurement to allow comparison or having a full scope of different measurables which can be applied to real world practice is key here.	

	This should be slightly easier to quantify and would encourage departments to move away from piped anaesthetic gases (ie using cylinders of nitrous as opposed to piped). If using piped, we had difficulty being able to quantify our specific use in ED and there tends to be a loss from piped supply. Would be easier to quantify MDIs and gases form anaesthetic machines that are bought in set units.	Piped anaesthetic gases have not been included in this calculator, because anaesthetic gases are rarely used in ED and, when used, they are normally managed and ordered by the anaesthetics team rather than emergency practitioners.
	I would change the gas names to the generic ones (e.g., nitrous oxide), from the brand ones (entonox). There are also other ways to measure gases e.g., directly measuring the Litres used. Suggest adding this is as an additional 'data source', 'key data collected' and 'unit of data'	Gas names have been changed to generic names from the brand names.
Electricity	OK for just electric energy. As previous how do we factor solar into this - solar on the roof of our hospital.	We decided not to include the use of green energies in the calculator, as they are already applied in the conversion factors provided by DEFRA.
	Possibly this should be combined with the energy section. Most departments will have a figure for gas and electricity, but this will be the trust average	This section has been separated from the energy section, as one represents direct emissions from the site and the other represents indirect emissions.
	I believe trying to quantify exactly what energy is used by a department will be difficult. When asking our trust the departments did not have set meters for different areas, though I assume this would be possible. Think all the options would be appropriate but unsure if they would be accessible.	The approach used by Prasad et al. can be applied, according to which the total consumption of the site is multiplied by the department's surface and divided by the total surface of the site. This formula can provide an estimation of the department's consumption and subsequently its carbon footprint.
	Probably on a trust level rather than department level? Otherwise not measurable.	
	Similar to the gas bills, making this specific to ED use will be difficult. With the added difficulty that as EDs operate 24/7 I assume we use more electricity per square metre than other areas of the hospital	
	Again, how easy will be it be to isolate ED electricity use from general hospital use? Is it often the case that there are separate meters?	
	I think the relationship between hospital electricity and ED electricity as a proportion of this needs to be explicated, as per the energy Q	
	Great to have in but again may be difficult to get the specific amount used in the ED if they don't have a separate meter	
Catering/food	What are you looking to improve with respect to food? If you say your metric is total kg of food, it sounds like you're suggesting we should be feeding patients less!	This calculator aims to provide a carbon baseline assessment and identify areas of high carbon intensity or hotspots in ED to facilitate sustainable initiatives. Thus, catering should be included in the assessment, as it contributes to the carbon footprint.
	Don't know if weight of food specifically would be that helpful/possible. Looking at total number of meals etc would be easier to assess.	Since there may be differences between hospitals, we considered it would be preferable to count the weight.
	Very broad here. Number of meals doesn't correlate with kg at the end so need to be consistent in the data value again here?	This has been revised and "number of meals" has been changed for "weight of meals".
	Instead, I would suggest a couple of obvious ones around food: increase in plant-based food options; reduction in plastic packaging. There also needs to be something based on reduction in single-use plastic utensils, plates, cups etc	This is an intervention rather than a measurement of the baseline carbon footprint of the ED, which is the aim of this calculator.
	"PLant based meals /menus? Food waste recycling. /Plant based milk use? Packaging of meals. - all more environmentally focussed rather than just number of meals - the patients	

	Just having number of meals for me is meaningless from an environmental perspective?"	
	I would potentially add £ as a unit of data. Also, annual expenditure may be an option for data source. And for 'key data' things like milk/juice, teabags and snacks (e.g. fruit, yoghurt) won't be covered by 'meals provided to patients' so I suggest expanding this section.	The financial cost and annual expenditure to measure this category was considered, although the researchers noted that the ordering process for catering differs between hospitals and is not homogeneous across the NHS.
Disposable/consumables	This is a massive area and should be broken into some of the key items: PPE, cannulas, single-use surgical instruments	This category has been divided into different sections to facilitate its navigation.
	Feel units of data could be in number of units (ie how many gloves etc) as opposed to kg. As most consumables will have different carbon footprints depending on their production and materials they are made of.	
	Once again this is a very diverse list to to be truly comparable and measurable each of the items will need a reliable unit of measurement. Kg isn't ok for some of them - eg cannulas? And also to convert from kg to Co2e for cannulas? would it not be units of cannulas used and then this is converted to kg/co2e? Detail is key here to enable measurement and conversion	
	I would definitely add £ / annual expenditure to the unit of data and key data sections, respectively! This procurement data is easy to get	
	This would obviously be a massive area. Should be able to get figures for most things from procurement figures but would need many categories to assess everything used in ED. Could possibly split into admin/clinical/catering/hygiene etc? This would be a massive body of work to undertake and items used would differ between departments and depend if ordering was done by the individual department or at trust level.	
	May need to include materials consumed (ie plastic, metal, paper). Pounds spent on consumables could be used if taking a top down approach	A top-down approach has been used for this category, based on the financial cost.
Medical equipment	Again, unsure if weight is best form of unit of measurement.	The weight of most of the medical equipment items is available and DEFRA provides a conversion factor for medical equipment based on weight, thus we considered it would be more accurate to measure this category per weight.
	Personally I'm not sure this rings well with me. There are so many types of medical equipment that this will be very tricky to deliniate. Again kg's - I think I might have misread this, but does that mean weighing the obs machines, uss machine etc?!	
	Again May need to consider pounds spent if using a top down approach.	
	Converting these to kg again will be a big task	
	Suggest adding £ as per above	
Non-medical equipment	As above, using number of units as opposed to weight.	The weight of most of the non-medical equipment items are available and DEFRA provides a conversion factor for this non-medical equipment based on weight, thus we considered it would be more accurate to measure this category per weight.
	Suggest adding £ as per above	
	Where does computer efficiency come in - i.e low power mode and screen brightness?	This is outside the boundaries of our calculator and has not been included.
Pharmaceuticals	This is a key one and hasn't been measured at all before. I think Greener NHS is trying to put together information regarding the footprint of individual medications so that will be a good source of information for the calculator.	

	Could break down into those used in emergency department, those given out as TTAs and look between IV/oral in those drugs that have 2 forms. Have found reducing things like IV paracetamol an easy win as save lots of unnecessary waste.	This has been considered and this category has been divided into different sections to facilitate its navigation.
Transport	I like the metric of km travelled - maybe in a 24 hour period, or day period. I.e a snapshot survey to record how many km total was travelled to get to /from the ed on that day or half day - and break down into staff groups etc. and modes - public transport/active travel/car commute. THis would need careful consideration and have a moderator for number of users etc. But could be very good.	Positive review. Nil changes made.
	I think this would have to be a snap shot or a sample size to get enough survey responses for accuracy	
Waste	Key data collection would be great I just wonder how feasible a full years worth of collection is? Maybe a few weeks and multiply up? Just a big commitment if done by clinical staff. Appropriate as a way of measuring if possible to collect.	Data can be collected through auditing over a period and multiplying for 52 weeks, obtaining the weight of each type of waste.
	kg or tonnes the measure here. We have done this and it is tricky but I think the only way. Note recycling is generally lighter than other waste so demonstrating good enviro change is tricky because the light weight doesn't change much. Annual waste in kg would need to be factored up as measurements can only really be done over 12 hours or thereabouts to make it practically possible.	
	I think the weight would be more in tonnes given amount of waste if it's done in the aggregate - suggest giving this as an option	This has been included.
Water	All appropriate, will just depend on the trust as to whether you can get individual data for the department or not.	The approach used by Prasad et al. can be applied, according to which the total consumption of the site is multiplied by the department's surface and divided by the total surface of the site. This formula can provide an estimation of the department's consumption and subsequently its carbon footprint.
	Can anyone really measure this on a departmental level?	
	Again this may be tricky without a meter for the ED itself.	
	Similar to gas and electricity, this may need a monitoring system in place for a week to find out the accurate amount of water the ED uses	
