# Supplementary material for: The emergency department carbon footprint calculator: Design and validation
Source: Medicine (Baltimore). 2025 May 30;104(22):e41652. doi: 10.1097/MD.0000000000041652 (PMC12129524; doi:10.1097/MD.0000000000041652)
Supplement: Supplementary file 2 [file medi-104-e41652-s002.pdf]

Supplementary Table 2. Comments on expert panel II.

Scope	Comment	Response
Scope 1	Total field should have Kg CO2e as units.	Kg CO2 added as unit in the total fields.
	What is the purpose of Hospital Floor Space and Emergency Department Floor Space columns in the Energy Consumption columns? Would WTT be included here even though we are looking at scope 1 emissions?	Hospital floor space and emergency department floor space are necessary to make an assumption of the energy used by the emergency department. Option A and B added. Option A when submeters for ED as available and option B when submeters for ED are not available, also added in electricity and water consumption. Well-to-tank (WTT) emissions are included in this category.
	Calculations are not correct in the anaesthetic gasses section, I.e., 200 cylinders of EA entonox would have a much bigger carbon footprint than 191.67 kg. Change column C to number of cylinders used in the past year, rather than in the department currently.	Error in calculation detected and changed.<br>Column C has been changed from “measured” to “used”
	Looks comprehensive. Probably just something I have missed but are all these quantities for one year? I think we would struggle to get accurate data of kms for hospital transport, but something I would be interested to look into. For our department this would also include things like blood products as these are often brought across by van in emergencies, just possibly something to add in?	Calculator is set for a year, clarification added in the information sheet and several cells of the calculator.<br>We recognise the challenges of monitoring transport; however, this can be attained by using a survey-based systematic approach.<br>Couriers and freight transport are outside of the boundaries of our calculator, as these services are often controlled by the hospital rather than by the emergency department, and thus obtaining the data specifically for the emergency department might not be feasible.
Scope 2	May need to include T+D and WTT	Transport and distribution (T+D) and Well-to-tank (WTT) emissions have been integrated in our calculator.
	I think this is a reasonable way to measure this. Some hospitals may be able to give more direct figures but useful to include even if an assumption, makes more of a case for turning equipment/lights off etc,	Positive review. Nil changes made.
Scope 3	Is the price at the Royal Free Hospital the same as the NHS supply chain? We should be using the price from supply chain for wider use. We should advise users to look at how many of these products they have used or ordered over a set period (ie a year), rather than the number currently in the emergency department, otherwise it only gives a cross section of the carbon footprint, whereas what we really want is a carbon footprint over a certain period. Change the title of Column C to ‘Quantity used’ over 12 months	We have compared and confirmed that the prices obtained from the Royal Free hospital are the same as the prices provided by the NHS supply chain catalogue.<br>The calculator is set for a year. Clarification added in the information sheet and several cells of the calculator.<br>Colum C changed from “quantity measured” to “quantity used”
	Some units missing for Conversion factors. Would benefit from being able to put in an 'Overall Spend' field, ie say for pharmaceuticals, where the emissions factor is the same throughout the different drugs. This would save time for EDs which don't have time to do a complete audit of all their medicines. Throughout the calculator, it should not look at the Quantity in Department, but the Quantity Used by the Department, over a certain time period.	Units reviewed and added to all conversion factors. “Overall spend field” added for pharmaceuticals and disposables, given the user option A -to measure carbon footprint item by item- or option B -to measure carbon footprint based on the overall expenditure.<br>“Quantity measured in the department” changed for “quantity used in the department”.
	Surprised to see the cannula conversion factor is so low. We got a much bigger CO2e in our cannula reduction project	The conversion factor has been revised and we think the mistake might be that the value shown in the calculator is per pack rather than per item. We have included the number of items per pack for each item.

	Do the transport emissions include WTT. These should be included. (Can be found in WTT - pass vehs&travel-land'.	WTT emissions have been added to transport emissions.
	Does this include patient/staff transport- both or one or the other? It should specify which is included.	This a decision to be made by the user collecting data rather than by the calculator itself.
	The units are wrong here. For example, 1000kg of domestic waste leads to only 1.72kg of CO2e, but it should be 172kg of CO2e per tonne. Also I thought from Chantelle's paper, that Offensive waste was 569kg per tonne, but you have 2.49 (again out by factor of 1000)	The units have been revised and an error in calculation has been detected – correction made.
	This is an unbelievable amount of work and looks very thorough. Seems to include most things I can think of. I guess there will be some differences in the catering between trusts. And wonder if possible, to include reusable vs disposable? Whether that would show the differences between the 2? Also, with things like suture packs, to show the difference between reusable and disposable, for things we are trying to change with Green ED?	Comparing reusables versus disposables will be a task to be completed after the carbon footprint assessment, as the carbon footprint of a reusable item will have to be divided by the number of uses.
	I notice that for the blood sample tubes it looks like only one type of system has been accounted for, is the carbon footprint of vacuettes vs vacutainers likely to be similar?	Differences between devices have been acknowledged. We have added a black cell in the disposables/consumables category for the user to be able to introduce items not available in the calculator.
Overall	Nothing I can think of, looks very comprehensive and think will be an invaluable tool.	Positive review. Nil changes made.
	In terms of patient/staff journeys to and from the department - is this outside the scope?	This a decision to be made by the user collecting data rather than the calculator itself.
	This tool will be powerful from an environmental perspective, and probably more so for the Trusts when combined with local financial data. Gathering feedback when it is in use might also lead to non-inferiority studies for alternatives to provide proof that lower carbon alternatives are also safe to use (e.g. oral/PR/IM alternatives to IV medications).	Positive review. Nil changes made.
